# An empirical study on cultivating college students' cross-cultural communicative competence based on the artificial-intelligence English-teaching mode

**DOI:** 10.3389/fpsyg.2022.976310

**Published:** 2022-12-06

**Authors:** Jingjing Long, Jiaxin Lin

**Affiliations:** ^1^Institute of Literature and Media, Dongguan University of Technology, Dongguan, China; ^2^School of Foreign Studies, Northwestern Polytechnical University, Xi'an, China

**Keywords:** artificial intelligence, English teaching, intercultural communication ability, comprehensive evaluation, intercultural sensitivity, fuzzy comprehensive evaluation

## Abstract

Artificial intelligence education will be an important part of information technology education in the future. It will enter the classroom and the learning life of students in various forms. The modern teaching media used in artificial intelligence courses at universities include some conventional teaching courseware, multimedia videos, and tool software. Some teaching media are unique to artificial intelligence, such as smart software, smart devices, and smart websites. The existing cross-cultural competence dimensions and evaluation scales were theoretically explained with factor analysis by comparing and selecting comprehensive evaluation methods in the work. Its main advantages were as follows: simple mathematical models and easy operation. The construction of a comprehensive evaluation model for college students' cross-cultural competence included the principles and ideas of model construction, the methods and steps of model construction, and model calculations. The survey results showed that the four-level scores of all samples had a significant positive correlation with foreign cultural knowledge, attitudes, and cross-cultural communication skills at 0.01 level (bilateral) and 0.05 (bilateral), respectively.

## Introduction

The excellent spiritual qualities in national cultures can be combined with modern thoughts after dynamic interpretation to function in forming the national spirit. The stereotype of national culture is a group consciousness established by social conventions. This group consciousness is deeply rooted in people's minds. When using a second language to communicate with others, we are inevitably affected by the thinking way in our mother tongue. Using one's culture to evaluate others' behaviors inevitably affects communication.

The work aimed to combine the situation of college students from the perspective of Chinese culture. Based on the most influential ICC (Intercultural Competence) scale and ICC model, we have designed and constructed a localized ICC scale suitable for college students. College students were randomly selected as the research objects to prove the reliability and operability of the model. An empirical study on the cultivation of college students' cross-cultural communicative competence was performed based on the artificial intelligence English teaching model to contribute to cross-cultural communicative competence.

Pulverness et al. (2017) investigated the formation of students' intercultural communicative competence for linguistic and ethnic backgrounds using the target language. He evaluated models for developing student ICC in an English language learning environment and provided examples of teaching materials required for implementation. Hashemian and Farhang-Ju (2020) believed that INTERCULTURAL communicative competence (ICC) has been introduced into L2 theory and research. Participants are stronger in introversion, extraversion, intuition, sensation, and judgment types. Bagui and Adder (2020) focused on the in-depth study in 2020. Most students show negative attitudes, that is, their cultural competence is not enough to avoid cross-cultural conflicts in various literary lectures. Olagbaju (2020) regarded Nigeria as a unique nation-state with many cultures. Cross-cultural competence is instilled in the country's ethnic members and cultural identities through legislation. There is little success despite his efforts. Wang (2021) believed that linguistics teaching is affected by various factors, and the model he proposed has certain effects. Gao (2020) used controlled experiments. Idris (2021) aimed to have a comprehensive understanding of ICC and recommended some ICC-related competencies for EFL or Indonesian English teachers. Competencies involve language, sociolinguistics, discourse, intercultural competence, and intercultural awareness, all of which can help EFL or English teachers in Indonesia develop their intercultural competence and students' ICC (Chung and Son, 2020).

The innovations of the work are as follows: (1) The artificial intelligence course in colleges and universities was discussed. Modern teaching media included some conventional teaching courseware, multimedia videos, tool software, and some AI-specific teaching media, such as smart software, smart devices, and smart websites. (2) A set of dimensions and evaluation scales were established. The indices, selection, and weight relationship of the questionnaire are evaluated based on cross-cultural abilities, and feedback is sought from relevant experts.

## Methods

### Artificial intelligence teaching

**Figure 1:**
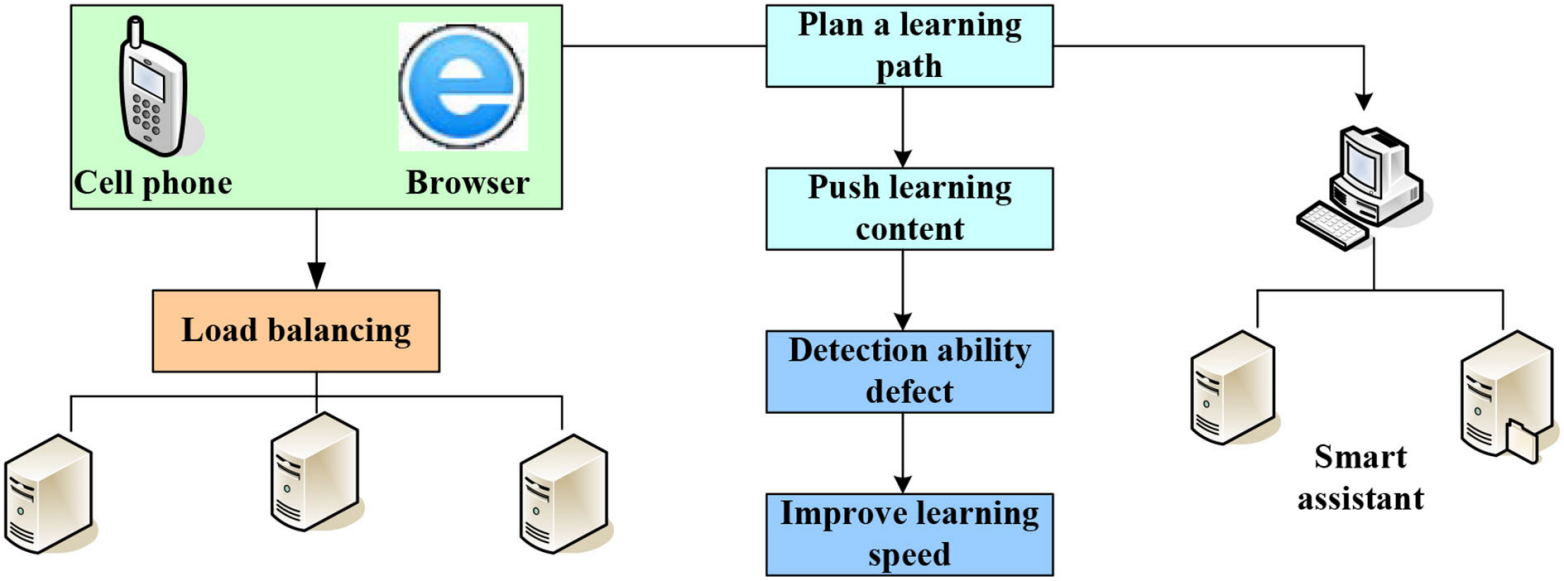
Artificial intelligence teaching.

Big data and intelligence in traditional fields have an inevitable development trend. The characteristics of the education industry are highly compatible with artificial intelligence in traditional fields. Therefore, the combination of artificial intelligence and the education industry is becoming a hot topic in artificial intelligence applications. At present, some related software and network resources for teaching artificial intelligence courses to college students have been gradually developed in China. However, website content is slow to update, reducing its usefulness. Therefore, the importance of website maintenance should be increased so that it has real opinions and is more suitable for artificial intelligence teaching. There are many learning tools on the Internet, such as search engines, online libraries, literature repositories, blogs, and Wikipedia for personal learning or online exploration teaching. There are not many blog resources for teaching reflections and teaching logs due to the small number of people currently researching artificial intelligence teaching. Network platforms have not yet played the most effective role in artificial intelligence education exchanges, which requires researchers to build such resources. Artificial intelligence teaching is shown in Figure 1. Artificial intelligence can solve several inherent problems in the field of education, such as teaching students following their aptitude, uneven distribution of educational resources, insufficient innovation ability training, and other problems. There will be new solutions to these problems in the age of artificial intelligence.

### Teaching media for artificial intelligence courses

Teaching media is the carrier of teaching content, the manifestation of teaching content, and the tool for transmitting information between teachers and students. The modern teaching media used in university artificial intelligence courses include some conventional teaching courseware, multimedia videos, and tool software, and some teaching media unique to artificial intelligence, such as smart software, smart devices, and smart websites. The following issues should be considered when choosing teaching media:

First, the integration of teaching media and teaching content is important. Multimedia videos, tool software, and courseware demonstrations are used in artificial intelligence teaching. Different media forms are used in different teaching contexts to complete different teaching tasks. For example, video teaching can be used to stimulate students' interest to avoid the uninteresting narrations of teachers. Tool software expert system shell Inter Modeler can be used in the teaching of expert systems.

Second, the teaching media must be consistent with the teaching goals. Teaching media is used to ensure effective teaching. It can achieve teaching goals and complete teaching tasks through its applications. The realization of three-dimensional teaching goals requires certain teaching media in artificial intelligence teaching in universities, such as the use of “Jinshan Express” to explain machine translation. Students have experienced the benefits of using these media. Artificial intelligence stimulates students' love for artificial intelligence and realizes 3D teaching in application fields. The 3D teaching mode emphasizes the theoretical basis of modern education theory and modern ideological and political education theory as well as the concept of people-oriented and student-centered teaching.

Third, teaching media should conform to students' cognitive characteristics and learning needs. It is not necessary to choose complex teaching media. Traditional teaching media generally refer to blackboards, chalks, textbooks, etc. Modern teaching media mainly refers to electronic media, which consists of hardware and software. When a certain type of knowledge can be presented in different forms of teaching media, teachers should consider which expression form is simple and suitable. For example, the presentation and use of intelligent software should try to choose what students are familiar with and life-related in college artificial intelligence courses. It can eliminate students' mystery of artificial intelligence technology and promote students' understanding of artificial intelligence. In short, the application of modern teaching media in the classroom is natural, and its selection should be based on effects that cannot be achieved by books or blackboard writing. A fuzzy uncertainty rule relationship is established (Duisembekova, 2021).


(1) $$\begin{array}{l} \mu =\left\{\mu_{\text{y}}\cdot W\right\} \end{array}$$

where μ_y_ represents the quantified vector of the inference conclusion. μ_p_ is assumed to be a weighted combination (Lei and Soontornwipast, 2020).


(2) $$\begin{array}{l} \mu_{\text{p}}=\overset{n}{\underset{\text{i}=1}{\sum }}W\cdot \mu_{y} \end{array}$$

The knowledge representation form after adding the weighting factor value into consideration is as follows (Hoff, 2020).


(3) $$\begin{array}{l} A_{M}=A_{1}\left(M_{1}\right)\wedge A_{2}\left(M_{2}\right)\wedge A_{3}\left(M_{3}\right)\wedge \ldots \wedge A_{n}\left(M_{n}\right) \end{array}$$

Supposing the credibility of each sub-condition is CF(A) (Harper, 2020),


(4) $$\begin{array}{l} \text{CF(A)}=\overset{n}{\underset{\text{i}=1}{\sum }}w\times CF\left(A_{i}\right) \end{array}$$

If the normalization conditions are not met (Ma, 2017), then


(5) $$\begin{array}{l} \text{CF(A)}=\overset{n}{\underset{\text{i}=1}{\sum }}w\times CF\left(A_{i}\right)/\overset{m}{\underset{\text{j}=1}{\sum }}w \end{array}$$

Then the credibility of conclusion B is as follows (Medeiros et al., 2021).


(6) $$\begin{array}{l} CF\left(B\right)=CF\left(B,A\right)⊗CF\left(A\right) \end{array}$$

(7) $$\begin{array}{l} B\left(X\Rightarrow Y\right)\\ =|\left\{K:M\cup N\subseteq K,K\in M\right\}|/|\left\{T:M\subseteq K,K\in U\right\}| \end{array}$$

### Evaluation methods and tools of cross-cultural competence

The culture of the physical form is mainly manifested in the forms of transformed natural objects, artificial products, and human beings, and the culture of the conceptual form is mainly manifested in the forms of art, morality, religion, political and legal thought, and language. The cultures of the physical form and concept form all penetrate and embody a common thing, and both involve the basic spirit of cultures—infiltrating all forms of culture and most important values that play a dominant role. The spiritual culture at the ideological value level is the core. Spirit is the core and life of cultures, and it is the deepest thing in cultural phenomena.

In recent years, the main evaluation methods used in the studies of cross-cultural competence can be summarized into the following three categories, indirect evaluation, direct evaluation, and mixed evaluation.

(1) Indirect evaluation of cross-cultural competences

The ISCI scale (the Intercultural Sensitivity Inventory) uses self-reporting to evaluate the ability of individuals to communicate and explain their behaviors in intercultural contexts. The BASIC scale (Behavioral Assessment Scale for Intercultural Competence) is used by researchers to evaluate an individual's intercultural competence based on his/her behaviors and analyze certain aspects of the individual's intercultural communication skills, such as respect for others, knowledge orientation, and empathy. This model mentions the description of skills, knowledge, attitude, and other standards and puts forward elements that other scholars have not been involved in, such as respect for others, task role behaviors, fuzzy tolerance degree, and interactive attitude'. Besides, certain language skills, textual skills, and social language can reflect whether there is a good attitude toward the culture of other countries. Therefore, it is important to master certain communication skills.

The Intercultural Development Inventory (IDI) is based on the cross-cultural sensitivity model and proposes a cross-cultural competence development scale with 44 descriptive items. It is mainly used to evaluate the intercultural competence development of individuals or groups level.

Intercultural competence is the basic competence of modern youth and the basis for civilized dialogue. Most existing indirect assessment tools include self-reporting mainly in the form of questionnaires, and they emphasize the overall structure of intercultural competence rather than the overall structure of intercultural competence.

(2) Direct evaluation of cross-cultural competences

Indirect evaluation is widely used, which largely reflects the time-consuming nature of direct evaluation data collection and analysis. However, these methods are more likely to provide more comprehensive information for evaluating cross-cultural competence because they can provide more detailed, subtle, and personalized explanations. Indirect evaluation or self-report evaluation methods discussed above are avoided.

Direct methods for evaluating intercultural competence include performance evaluation, learning profile evaluation, and interviews. Their common ground is the discovery of cross-cultural competencies that individuals exhibit in their behaviors whether in the actual context (performance evaluation), reflection and accumulation at work (learning profile evaluation), or one-on-one communication with interlocutors (interview).

(3) Mixed evaluation of cross-cultural competences

The mixed use of indirect and direct evaluation methods can reveal more objective laws and changes in the development of evaluated cross-cultural competence rather than the subjective results of the indirect self-evaluation of cross-cultural competence.

### Dimensions of cross-cultural competence evaluations

The work uses Fantini's cross-cultural competence evaluation dimension.

Native language level: communication and communication skills in the native language.

Level IV: Cross-cultural and multicultural experts (suitable for teachers and educators who are engaged in the training, education, consultation, or advice of multinational students). Table 1 shows the confirmatory factors of the fit indices of the ICC evaluation scale for college students.

**Table 1:** Confirmatory factors for the fit indices of the ICC evaluation scale for Chinese college students.

**Index**	**Reference value**	**Index value**
NFI (normal adaptation index)	>0.80	0.82
CFI (comparative fit index)	>0.90	0.92
NNFI (non-standard fit index)	>0.90	0.91
GFI (fitness index)	>0.90	0.91
AGFI (adjusted fitness index)	>0.90	0.93
RMR (mean square and square root of residual errors)	<0.08	0.07
RMSEA (asymptotic residual mean square and square root)	<0.08	0.03

### Fuzzy comprehensive evaluation of cross-cultural competence

**Figure 2:**
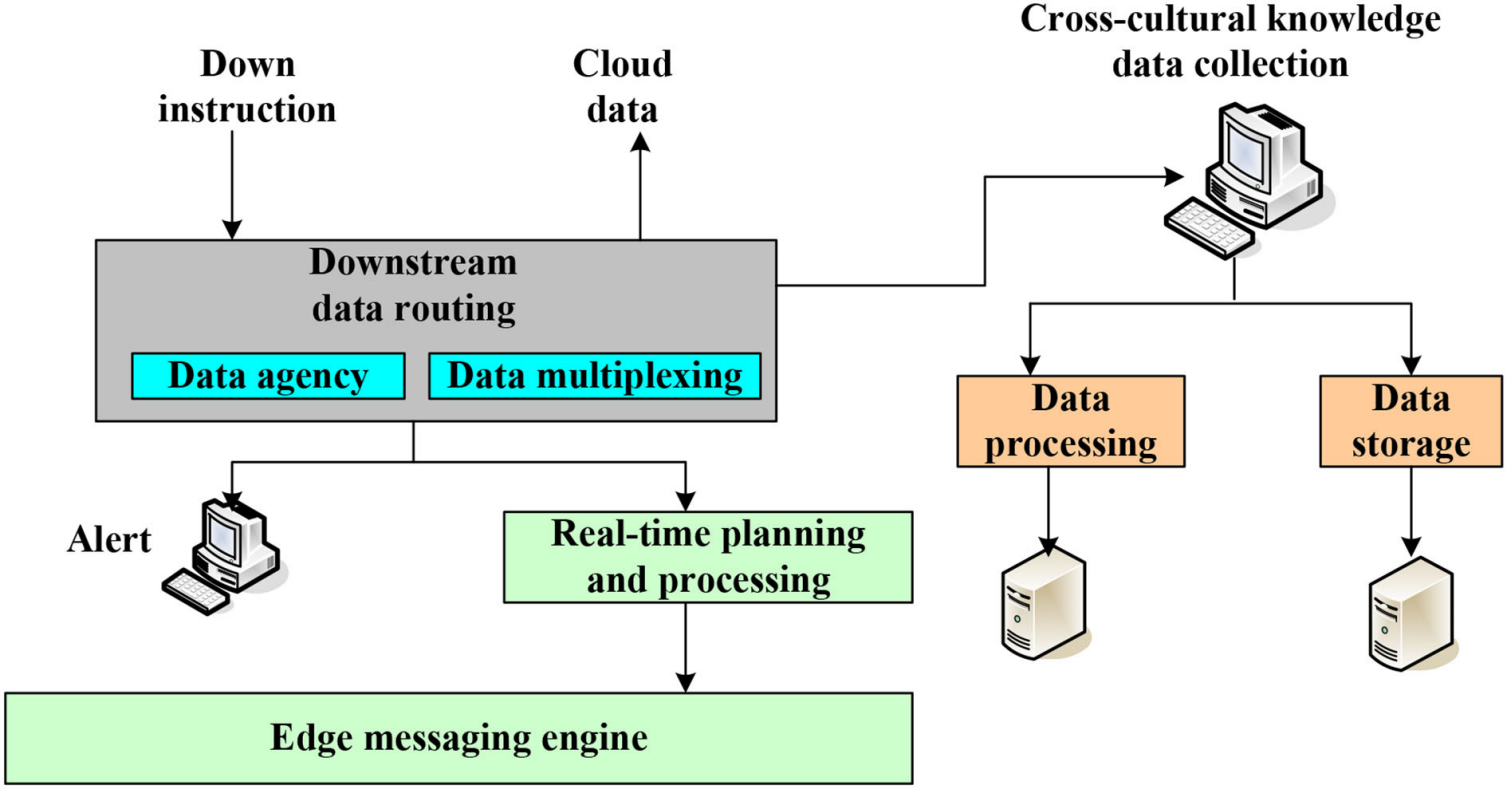
Wisdom teaching of cross-cultural competence.

Current research is relatively fragmented, with few quantitative empirical studies and a lack of systematic and comprehensive evaluation. Therefore, the work uses the fuzzy comprehensive evaluation method and systematic comprehensive of college students' cross-cultural competence. The fuzzy comprehensive evaluation method is a comprehensive evaluation method based on fuzzy mathematics. This comprehensive evaluation method transforms qualitative evaluation into quantitative evaluation according to the membership degree theory of fuzzy mathematics, that is, using fuzzy mathematics to make a general evaluation of things or objects restricted by many factors. It has clear results and strong systematicness, which can solve vague and difficult-to-quantify problems and various non-deterministic problems. A systematic comprehensive evaluation is to use models and various data to conduct an overall and comprehensive evaluation of the program from the overall point of view of the system based on the technical evaluation, economic evaluation, and social evaluation of the system program. Cross-cultural competence is a complex combination of competencies composed of four main dimensions, knowledge, attitude, skills, and consciousness, concerning research results of cross-cultural competence. Figure 2 shows the teaching of wisdom and intercultural competence.

A localized evaluation scale and evaluation scale are constructed, including domestic cultural knowledge, foreign, attitudes, communication skills, cognitive skills, and awareness. The existing comprehensive evaluation methods are screened through comparison. Its main advantages are as follows: the mathematical model is simple, and the operation is convenient. Moreover, practical application in cases has proved that the fuzzy comprehensive evaluation method has high reliability and validity, convenience, flexibility, and easy operation. The use of the fuzzy comprehensive evaluation method can obtain a comprehensive and reasonable convincing evaluation. There are many traditional comprehensive evaluation methods, but none of them can be suitable for various places and solve all problems. Each method has its focus and main application areas. The fuzzy synthesis method is more suitable for new problems in a new field.

The general steps of a fuzzy comprehensive evaluation of college students' intercultural competence are as follows. Determine the set of factors for evaluating intercultural competence, the set of comments for intercultural competence evaluation, the weight distribution between evaluation factors, and the fuzzy evaluation transformation matrix of intercultural competence (Li and Wang, 2020). Then, establish a fuzzy comprehensive evaluation model of grade and grade two multi-factors, and modeling Liu et al. (2021):


(8) $$\begin{array}{l} V=\left(v_{1},v_{2},v_{3},...,v_{m}\right) \end{array}$$

Number *m* of evaluation levels is usually between 4 and 9. Here *m* = 5 (Xie and Mai, 2020).


(9) $$\begin{array}{l} V=\left(v_{1},v_{2},v_{3},v_{4},v_{5}\right) \end{array}$$

That is, *V* = (very weak/slightly, weaker/a little bit, average/some, stronger/more, very strong/very much) (Gaobin et al., 2020).


(10) $$\begin{array}{l} A=\left(a_{1},a_{2},a_{3},...,a_{n}\right) \end{array}$$

where *A* is the weight distribution set. The evaluation data of 20 domestic experts on the importance of various indices are collected for evaluating college students' cross-cultural competence and statistical processing. Then, the weight coefficients of the following indices are obtained according to the Delphi method. The weight of the first level index (Koenig et al., 2020) is:


(11) $$\begin{array}{l} A=\left(a_{1},a_{2},a_{3},a_{4},a_{5}\right)=\left(0.05,0.3,0.19,0.25,0.06,0.15\right) \end{array}$$

The secondary index weight (Liang, 2021) is:


(12) $$\begin{array}{l} A_{1}=\left(a_{11},a_{12},a_{13}\right)=\left(0.33,0.27,0.4\right) \end{array}$$

(13) $$\begin{array}{l} \;A_{2}=\left(a_{21},a_{22},a_{23},a_{24},a_{25},a_{26},a_{27}\right)\\ =\left(0.18,0.18,0.2,0.14,0.12,0.06,0.12\right) \end{array}$$

(14) $$\begin{array}{l} A_{3}=\left(a_{31},a_{32},a_{33}\right)=\left(0.5,0.17,0.33\right) \end{array}$$

The sum of weighted components is generally required to be 1 for fuzzy calculations (Liang, 2021).


(15) $$\begin{array}{l} \;A_{4}=\left(a_{41},a_{42},a_{43},a_{44},a_{45},a_{46},a_{47},a_{48},a_{49}\right)\\ =\left(0.09,0.18,0.15,0.12,0.16,0.06,0.12,0.13,0.05\right) \end{array}$$

(16) $$\begin{array}{l} A_{5}=\left(a_{51},a_{52},a_{53}\right)=\left(0.33,0.22,0.44\right) \end{array}$$

(17) $$\begin{array}{l} A_{5}=\left(a_{61},a_{62},a_{63}\right)=\left(0.35,0.42,0.34\right) \end{array}$$

The comment rating of each index is given through the judgment of each index, so the relationship between the evaluation index and the comment rating is established. Cultural-competence fuzzy evaluation transformation matrix *R* (Li et al., 2020; Nie, 2020) is as follows:


(18) $$\begin{array}{l} R_{\left(\text{j}\right)}=\left[{\vec{R}}_{1\left(\text{j}\right)},{\vec{R}}_{2\left(\text{j}\right)},{\vec{R}}_{3\left(\text{j}\right)},\ldots ,{\vec{R}}_{n\left(\text{j}\right)}\right] \end{array}$$

(19) $$\begin{array}{l} R=\left[{\text{r}}_{m\left(j\right)}\right]=\left[\begin{array}{cccc} r_{11\left(j\right)} & r_{12\left(j\right)} & \cdots & r_{1n\left(j\right)}\\ r_{21\left(j\right)} & r_{22\left(j\right)} & \cdots & r_{2m\left(j\right)}\\ \cdots & \cdots & \cdots & \cdots \\ r_{n1\left(j\right)} & r_{n2\left(j\right)} & \cdots & r_{nm\left(j\right)} \end{array}\right] \end{array}$$

where r_*m*(*j*)_ represents the membership degree of *R*. The fuzzy comprehensive evaluation model (Du, 2020) is:


(20) $$\begin{array}{l} B=A◦R=\left(a_{1},a_{2},a_{3},a_{4},a_{5},a_{6}\right)◦\left[\begin{array}{c} B_{1}\\ \ldots \\ B_{m} \end{array}\right]\\ =\left(b_{1},b_{2},\ldots ,b_{m}\right) \end{array}$$

where B is the comprehensive evaluation result of college students' cross-cultural competence U (Li et al., 2020).

## Results

**Figure 3:**
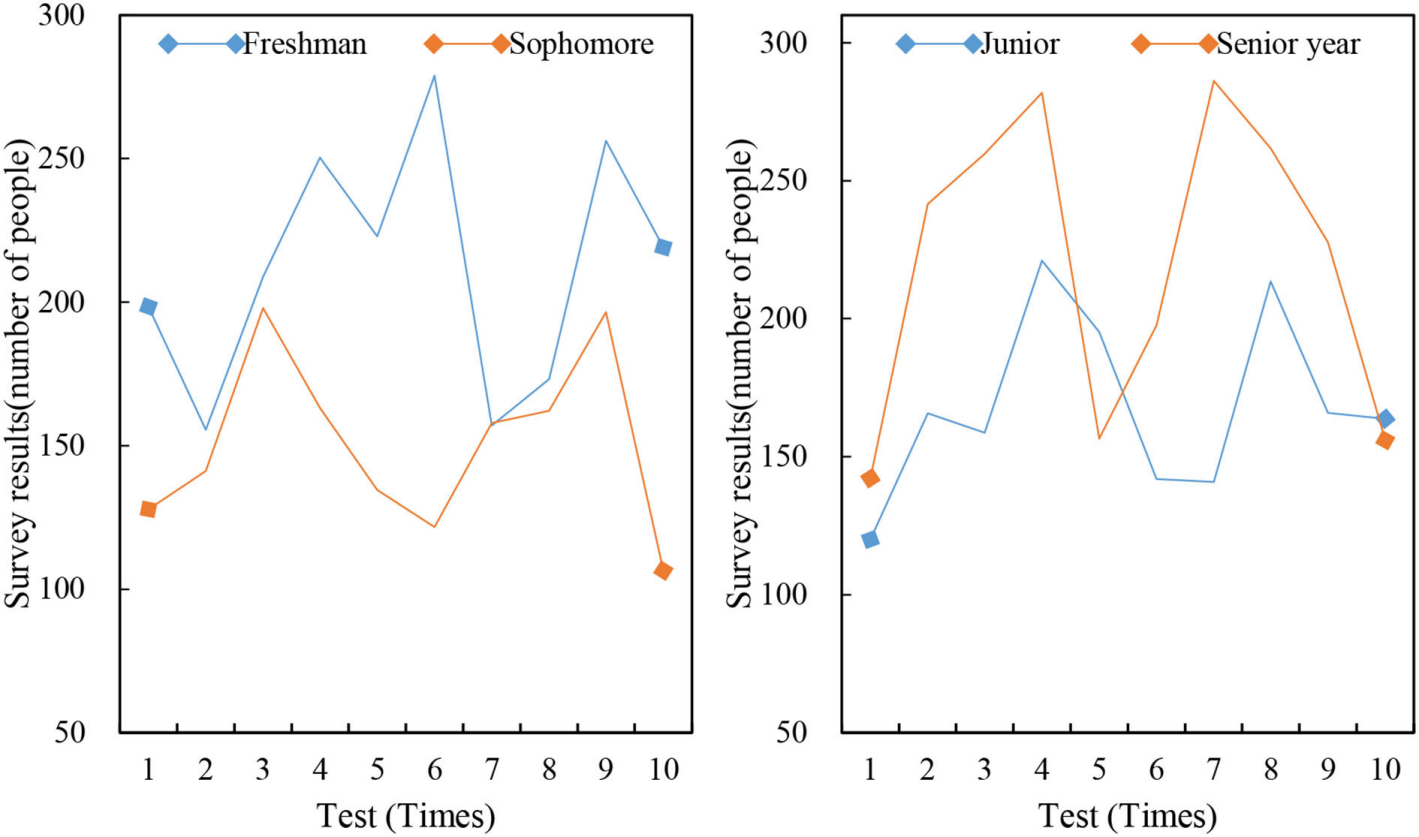
Number of freshmen to seniors.

A total of 1,300 questionnaires were sent out in the work, 325 for each grade, and 1,050 valid questionnaires were collected, including 313 for freshmen, 295 for sophomores, 232 for juniors, and 210 for seniors. Besides, 651 male students and 399 male students participated in the questionnaire survey, which accounted for 62 and 38% of the gender ratio, respectively. Two hundred eighty-three went abroad, which accounted for 27%. There were 112 foreigners from different cultures who had “a lot” contacts, which accounted for 11%. Seven hundred three had experience in cross-cultural communication activities, which accounted for 67%. Figure 3 shows the number of freshmen to seniors.

The Cronbach alpha coefficient value is tested for consistency (see Table 2 for test results). The overall Cronbach alpha coefficient is 0.9. The measurement results of this scale are reliable.

**Table 2:** Test results.

**Factor**	**Knowledge A**	**Knowledge B**	**Manner**	**Skill A**	**Skill B**	**Consciousness**	**Total table**
Cronbach alpha coefficient	0.734	0.91	0.863	0.873	0.779	0.878	0.9
Number of items	3	7	3	3	3	3	28

All the samples of 240 people are described from ICC comprehensive evaluation and ICC individual index evaluation: 38 people have a score of 600 or more in the fourth level; 170 people have a score between 490 and 599 points; 32 have a score of 489 or less.

A statistical analysis of college students was performed in the work. Two hundred and forty college students in the selected sample had a CET-4 score of about 550 and reached an excellent level. However, their intercultural competence was still at an average level. Therefore, foreign language teachers in addition to training students' language skills should teach more cross-cultural competence-related content. The results of descriptive statistical analysis are shown in Table 3.

**Table 3:** Descriptive statistical analysis results.

**Factor**	**Minimum**	**Maximum**	**Mean**
ICC comprehensive evaluation	0.17	0.50	0.5145
National cultural knowledge	0.01	0.05	0.0175
Foreign cultural knowledge	0.03	0.17	0.1075
Manner	0.07	0.17	0.1345
Intercultural communication skills	0.03	0.13	0.1355
Cross-cultural cognitive skills	0.01	0.05	0.0157
Consciousness	0.05	0.14	0.0771

**Figure 4:**
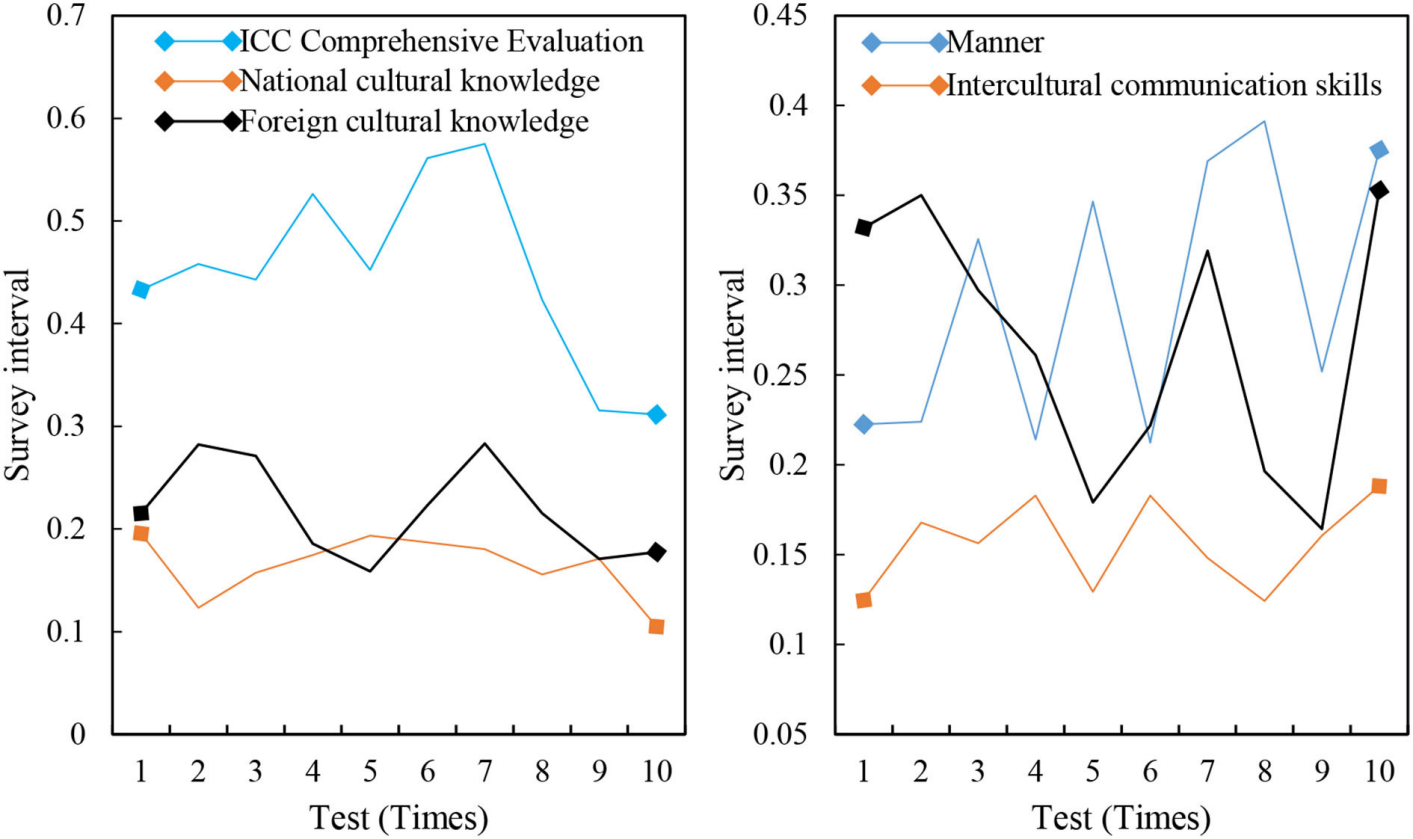
ICC comprehensive evaluation of 240 people.

The mean value of the ICC comprehensive evaluation of 240 selected students is 0.4950, and the reference value is in [0, 1]. The score of all samples in foreign cultural knowledge is 0.0982 in terms of individual index evaluation of each ICC, indicating that the foreign cultural knowledge of all samples is seriously inadequate and at a low level. However, the score of national cultural knowledge is 0.0285, and the reference value is in [0, 0.05]; the score of cross-cultural communication skills is 0.1304, and the reference value is in [0, 0.25]. The comprehensive evaluation of 240 ICC participants is shown in Figure 4.

The work found that 38 students with a score of 600 or more in CET-4 were subjected to the ICC comprehensive evaluation, and the comprehensive evaluation results of each index were similar to the overall situation of the 240 samples analyzed above. Besides, they have not developed their language skills with excellent foreign language CET-4 scores. The distribution of survey samples is shown in Table 4.

**Table 4:** Distribution of survey samples.

**Factor**	**Minimum**	**Maximum**	**Mean**
ICC comprehensive evaluation	0.28	0.90	0.5245
National cultural knowledge	0.01	0.05	0.0289
Foreign cultural knowledge	0.03	0.27	0.1076
Manner	0.08	0.17	0.1345
Intercultural communication skills	0.03	0.23	0.1395
Cross-cultural cognitive skills	0.01	0.05	0.0297
Consciousness	0.05	0.14	0.0871

**Figure 5:**
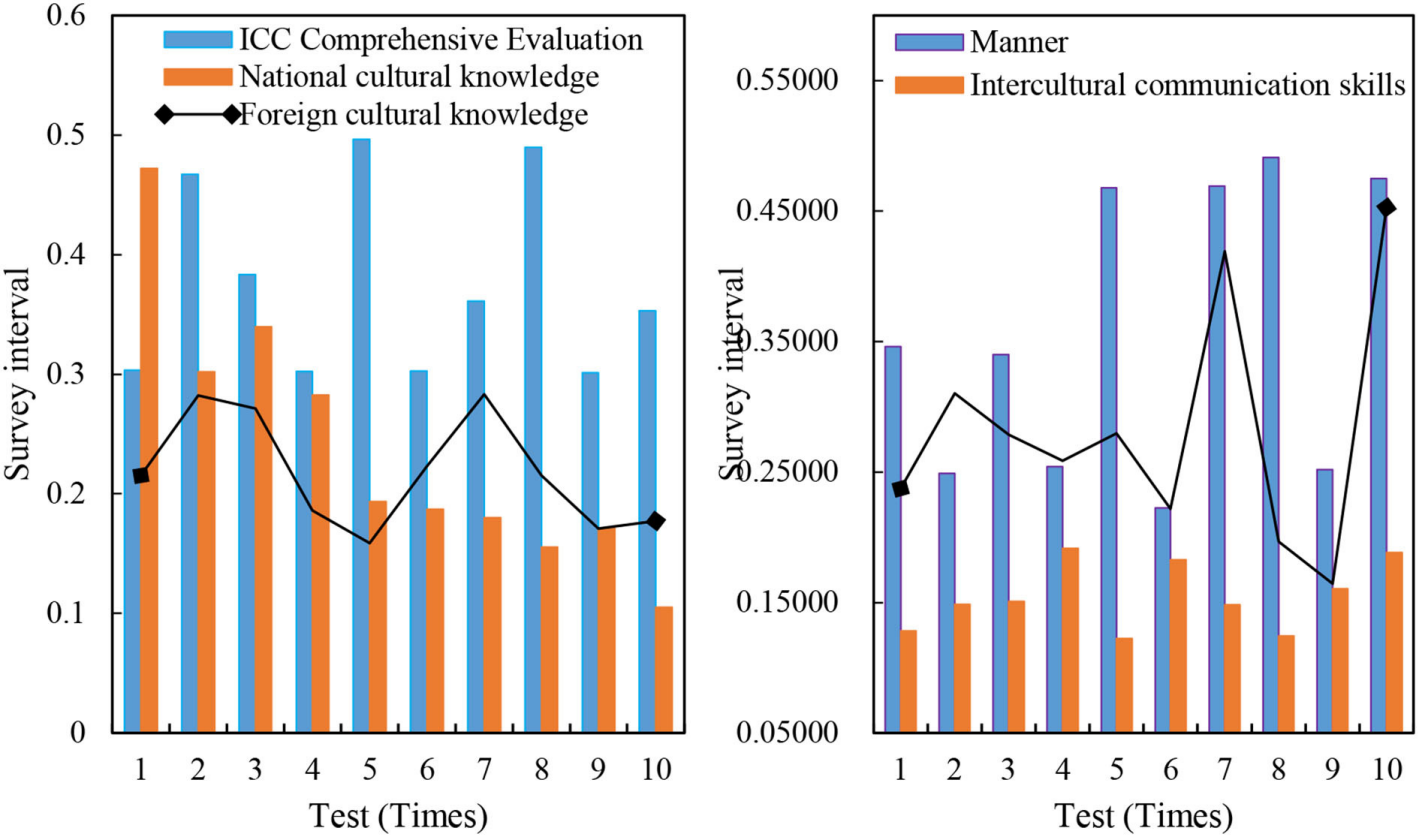
Evaluation result with a score of 600 or more in CET-4.

There are 38 people with CET-4 scores above 600. Their average ICC comprehensive evaluation is 0.5245, which is above 0.5, and their ICC is slightly higher than the average level. The foreign cultural knowledge score is 0.1076, and the reference value is in [0, 0.30]. The foreign cultural knowledge mastered by college students with a score of more than 600 in CET-4 is seriously lacking, and the score indices (history and geography abroad) of individual ICC are low; social political knowledge and basic knowledge of cross-cultural communication strategies are relatively weak. The national cultural knowledge score is 0.0289, the reference value is in [0, 0.05], and the reference value is in [0, 0.19]. On the contrary, the sample has a score of 0.1345 in attitude, and the reference value is in [0, 0.19]. The score is in the upper middle, indicating that college students with a score of 600 or more have a strong ability in cross-cultural attitudes and are at a higher level, such as the ability in cross-cultural flow willingness and interest, racism, and other communication barriers. The evaluation result of the grade 4 score of 600 points or more is shown in Figure 5.

There are 170 people with scores between 490 and 599. Their average ICC comprehensive evaluation is 0.4969, which is close to 0.5, and their ICC is at a general level. The score of foreign cultural knowledge is 0.0984, and the reference value is in [0, 0.30]. College students with a score of 490 to 599 have a very weak mastery of foreign cultural knowledge, and the individual ICC indices are scored. The value is also generally low. The comprehensive evaluation of 170 college students' cross-cultural competence is shown in Table 5.

**Table 5:** Comprehensive evaluation of cross-cultural competence of 170 college students.

**Factor**	**Minimum**	**Maximum**	**Mean**
ICC comprehensive evaluation	0.48	0.90	0.5445
National cultural knowledge	0.01	0.05	0.0489
Foreign cultural knowledge	0.02	0.48	0.1085
Manner	0.08	0.18	0.1245
Intercultural communication skills	0.02	0.42	0.1295
Cross-cultural cognitive skills	0.01	0.05	0.0498
Consciousness	0.05	0.14	0.0881

**Figure 6:**
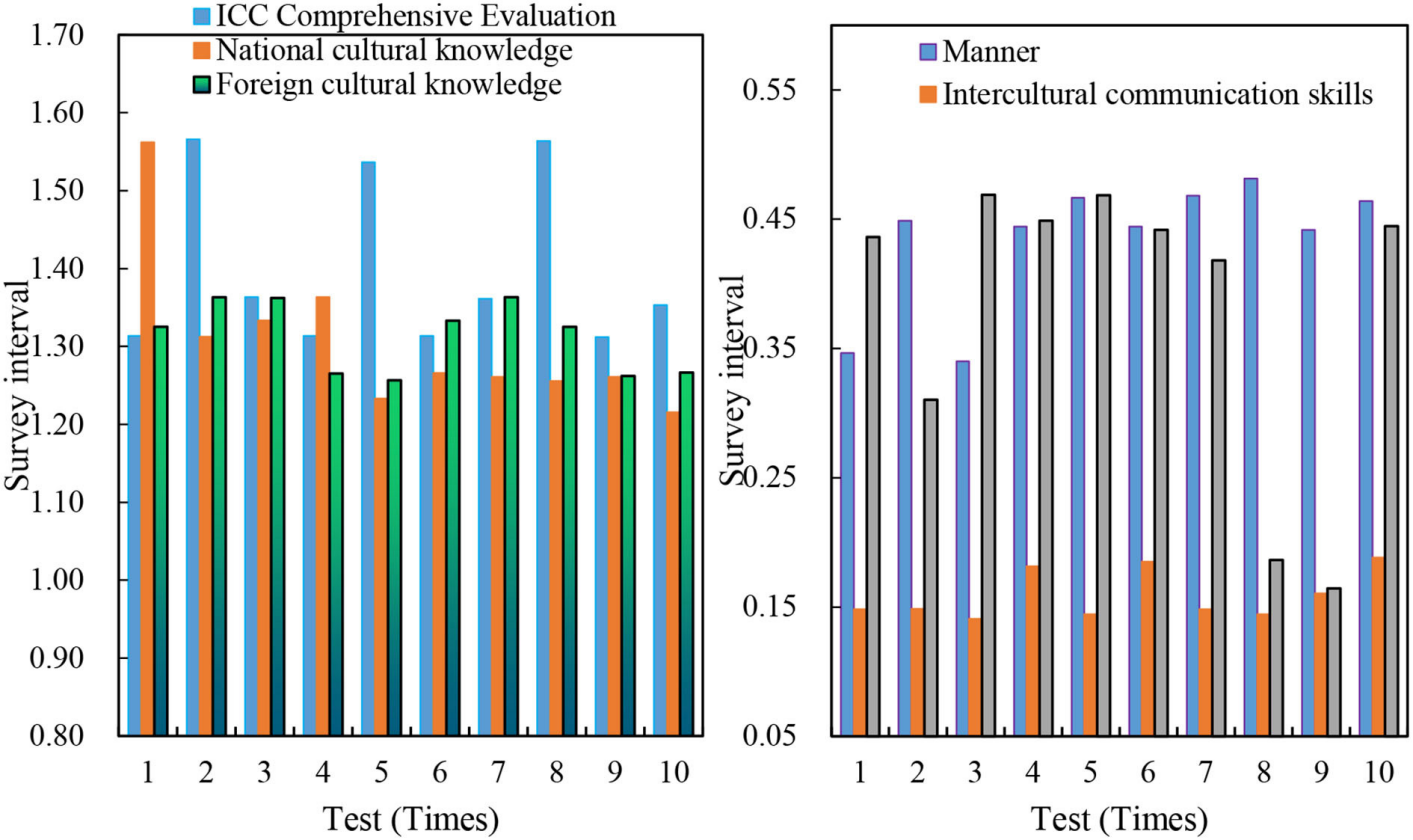
Attitudes of college students with a score of 490–599 in CET-4.

The sample's attitude score is 0.1291, the reference value is in [0, 0.19], and its attitude score is in the upper middle position within the reference value range. College students with a score of 490 to 599 have stronger intercultural attitudes ability. That is, the ICC level is higher, such as the ability in communication barriers such as willingness and interest in cross-cultural flow. Similarly, the ICC comprehensive evaluation of college students whose scores are between 490 and 599 is similar to the results of the 240-person sample analyzed above. Figure 6 shows the attitude of college students whose scores are between 490 and 599 in grade 4.

**Figure 7:**
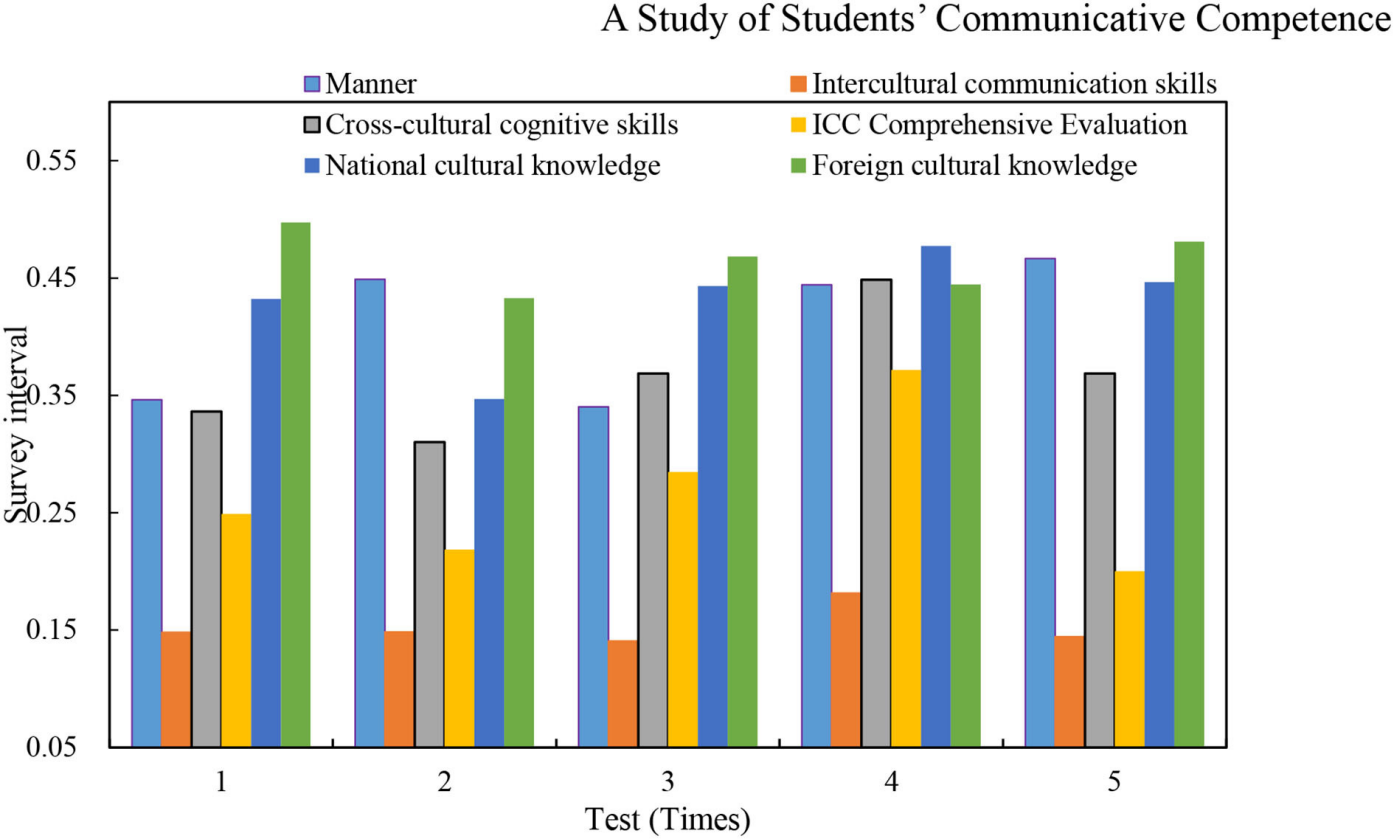
Evaluation of students with grades below 489.

There are 32 people with grade 4 scores below 489. Their average ICC comprehensive evaluation is 0.4494, slightly lower than 0.5, and their ICC is slightly lower than the general level. The score of foreign cultural knowledge is 0.0862, and the reference value is in [0, 0.30], indicating that college students with scores below 489 have a very weak mastery of foreign cultural knowledge, and the scores of each ICC single index are generally low. However, the score of domestic cultural knowledge is 0.0291 with a reference value of [0, 0.05]; the score of cross-cultural communication skills is 0.1178 with a reference value of [0, 0.25]; the score of cross-cultural cognitive skills is 0.0256 with a reference value [0, 0.06]; the score of consciousness is 0.0841, with a reference value of [0, 0.19]. The scores of the above four abilities are in the middle. The scores of the above four abilities are in the middle. The evaluation of students with a score of 489 or less in cet-4 in the sample is shown in Figure 7.

**Figure 8:**
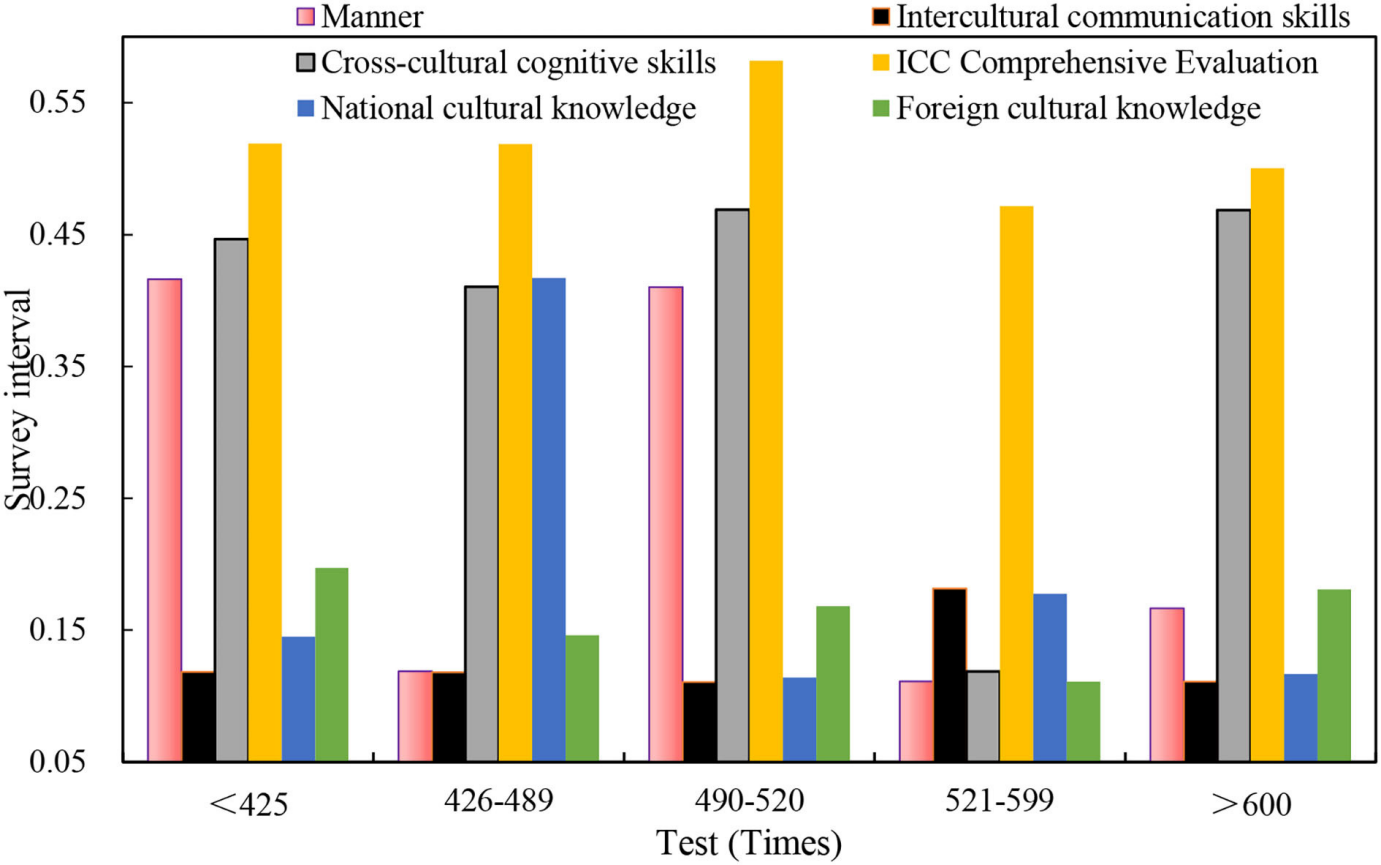
Impact of different CET-4 performance and cross-cultural communicative competence.

The calculation results of the ICC comprehensive evaluation model show that the ICC capabilities of selected samples are at a general level, and most people are seriously inadequate in foreign cultural knowledge. Ability, skills, and consciousness are in the middle. On the contrary, they have the strongest ability in attitudes. Moreover, CET-4 performance variables show that its ICC level also has a corresponding positive change with the positive change in performance. However, the fluctuation range is not large, and they are all at the middle level. Figure 8 shows the influence of different four-level performances and cross-cultural communicative competence.

**Figure 9:**
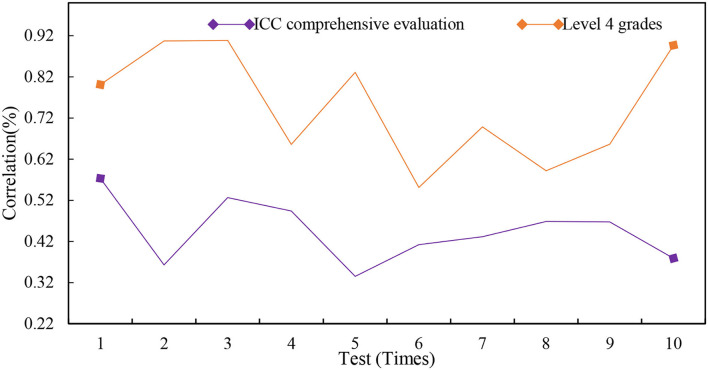
Significance evaluation.

The correlation coefficient between the CET-4 scores of 240 people and its ICC comprehensive evaluation score is 0.179, and it is significantly correlated at the 0.01 level (two-sided). There is an overall mutual promotion between the language ability of college students and their intercultural ability ICC. The significance evaluation is shown in Figure 9.

**Figure 10:**
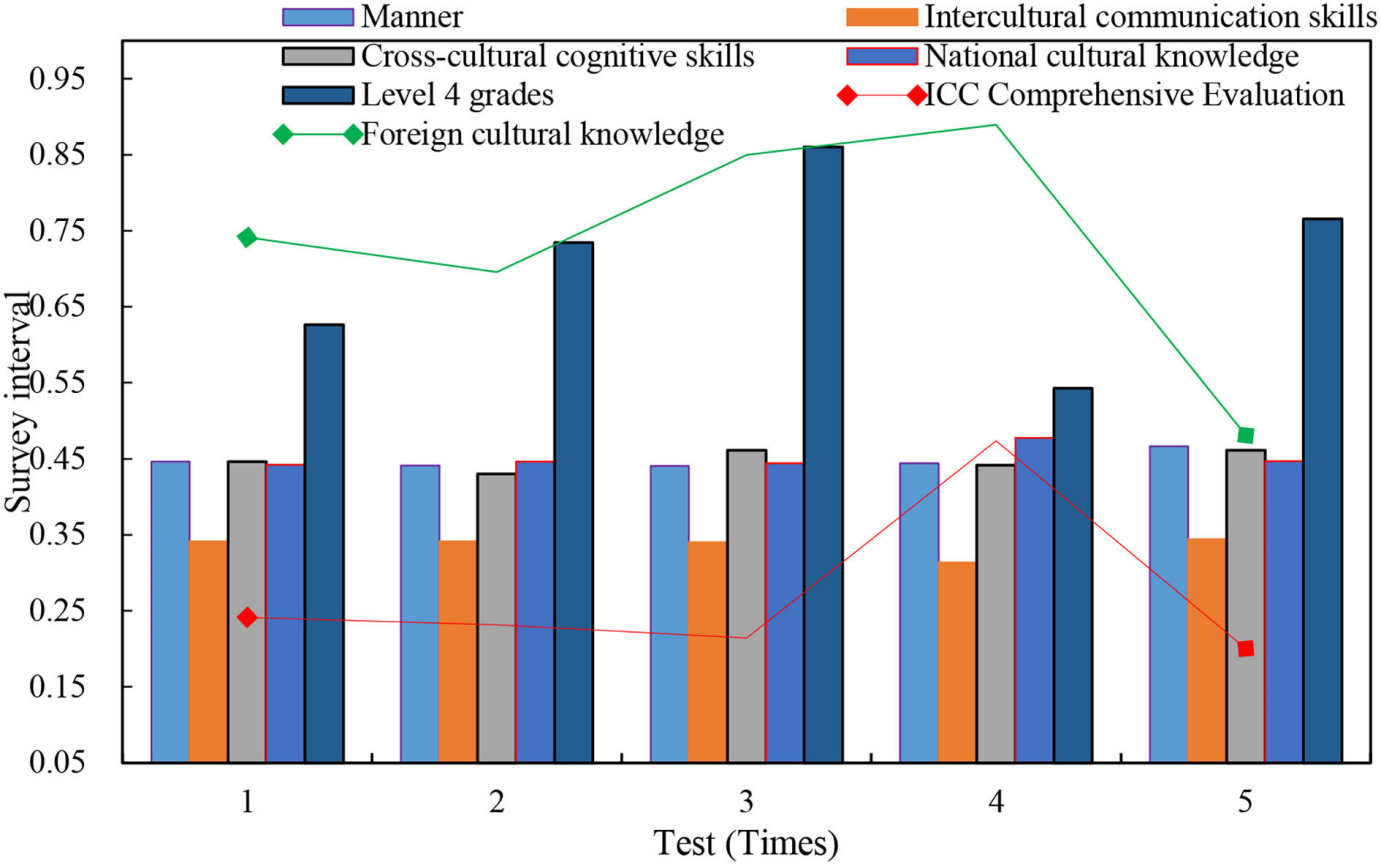
Correlation between the four-level performance and its ICC individual indices.

The correlation coefficients between the four-level scores of 240 samples and the individual indices of the ICC are as follows: cross-cultural cognitive skills of 0.111, awareness of 0.036, and the four-level scores of all samples and foreign cultural knowledge, attitudes, and cross-cultural communication skills of 0.01. There is a significant positive correlation between the level (two-sided) and the 0.05 level (two-sided). The correlation between the four-level performance and its ICC individual indicators is shown in Figure 10.

## Discussion

The stimulation of students' creativity in artificial intelligence teaching is important. Based on these principles and methods to achieve cross-cultural requirements, the performance evaluation concept in process evaluation is appropriately introduced so that teaching evaluation truly plays a role in regulating feedback and achieves the optimization of teaching effects. International market competition is becoming increasingly fierce with the increasingly accelerated process of globalization. they have encountered many difficulties and problems in the process of internationalization and higher education, e.g., cultivating international vision and international capabilities and evaluating the international capabilities of college students. Moreover, these issues have also become hot topics in research (Hou, 2020; Bai, 2021).

Scenarios for cultivating cross-cultural awareness and sensitive communication are as follows: initially denying the cultural differences between the two ethnic groups, not facing cultural differences, resisting and avoiding cultural differences, accepting and slowly weakening cultural differences, and finally contacting and involving cultural differences in cross-cultural communication. If teachers can have a clear understanding of the development stage of cross-cultural consciousness of learners at this stage and design teaching content and process based on this, they will surely achieve unprecedented success (Guo, 2020).

The reason why communication has become the research object of many disciplines is that it is inseparable from all aspects of human activities. Communication is regarded as a part of cultural studies. There are many fields involved in communication from the cultural perspective, and it is a fairly broad concept; therefore, the modes are diverse. Generally speaking, research involving social-cultural factors and communication can be regarded as the communication mode of cultural science. If the goal of cultivating high-quality foreign language talents is not empty talk, new teaching content, methods, and educational concepts are needed to guarantee and support.

Although we should try our best to constrain our words and deeds with the way of thinking and expression of the language we learn in the process of language learning, we must not lose our culture and blindly pursue the culture of the language we learn. Cross-cultural communication is two-way communication. It is completely unnecessary to lose one's culture. The language learned is accepted rationally and objectively under the premise of not losing one's culture. We should explore the constituent elements of cross-cultural communication ability after possessing a sense of cross-cultural communication and understanding which specific indices are achieved. If having the ability in cross-cultural communication, we can teach language and culture in a targeted manner and strive to enable students to complete various indices as soon as possible to truly have the ability in cross-cultural communication (Bin and Mandal, 2019; Gaobin et al., 2020).

## Conclusions

The questionnaire results were analyzed to study the current situation of cross-cultural sensitivity colleges and universities, the relationship between intercultural sensitivity and intercultural pragmatic competence, and the possible influence of intercultural sensitivity on intercultural pragmatic competence. The work explained the background and purpose of international talent training goals in universities. Secondly, a new research path reference was provided for researchers to study cross-cultural competence and analyze key factors (awareness, attitude, knowledge, and skills).

It can be used to evaluate the performance of international talents and has important practical significance to provide a powerful reference for evaluation methods. However, the work does not study cross-cultural communicative competence due to the limitations of time and technology. Further research is required.

## Data availability statement

The original contributions presented in the study are included in the article/supplementary material, further inquiries can be directed to the corresponding author.

## Author contributions

JLo contributed to writing and data collection. JLi contributed to data pre-processing. All authors contributed to the article and approved the submitted version.

## Funding

This work was funded by the Hunan Provincial Educational Teaching Reform Project (Grant No. HNJG-1239), Hunan Province Philosophy and Social Science Foundation Project (Grant No. 19WLH03), and Hunan Province Philosophy and Social Science Foundation Project (Grant No. 17).

## Conflict of interest

The authors declare that the research was conducted in the absence of any commercial or financial relationships that could be construed as a potential conflict of interest.

## Publisher's note

All claims expressed in this article are solely those of the authors and do not necessarily represent those of their affiliated organizations, or those of the publisher, the editors and the reviewers. Any product that may be evaluated in this article, or claim that may be made by its manufacturer, is not guaranteed or endorsed by the publisher.
